# Neurotoxic and some systemic effects of cadmium oxide nanoparticles following their subchronic intranasal administration to rats: experimental data analysis

**DOI:** 10.1007/s00210-025-04535-8

**Published:** 2025-09-06

**Authors:** M. P. Sutunkova, I. A. Minigalieva, L. V. Shabardina, I. G. Shelomentsev, A. K. Labzova, R. R. Sakhautdinova, L. V. Toropova

**Affiliations:** 1 Yekaterinburg Medical Research Center for Prophylaxis and Health Protection in Industrial Workers, Popov St., 30, Yekaterinburg, 620014 Russian Federation; 2 Ural State Medical University, Repin St., 3, Yekaterinburg, 620028 Russian Federation; 3 Laboratory of Stochastic Transport of Nanoparticles in Living Systems, Ural Federal University, Lenin Ave., 51, Yekaterinburg, 620000 Russian Federation; 4 Laboratory of Mathematical Modeling of Physical and Chemical Processes in Multiphase Media, Department of Theoretical and Mathematical Physics, Ural Federal University, Lenin Ave., 51, Yekaterinburg, 620000 Russian Federation; 5 Otto-Schott-Institut für Materialforschung, Friedrich-Schiller-Universität, Löbdergraben, 32, 07743 Jena, Germany

**Keywords:** Cadmium oxide nanoparticles, Intranasal administration, Toxicity, Neurotoxicity, Rats

## Abstract

Industrial emissions of cadmium, which is used in various industries, and formation of cadmium nanoparticles during high-temperature technological processes aggravate the problem of chemical pollution. Insufficient understanding of the toxic impact of cadmium nanoparticles, coupled with the serious multisystem toxicity of this metal, necessitate expansion of knowledge about their potential toxic effects. We assessed the response of female outbred albino rats to subchronic exposure to cadmium oxide nanoparticles (CdO NPs) administered intranasally at the concentration of 0.5 mg/mL thrice a week during 6 weeks. Adverse changes in the exposed animals found at all levels of organization in the body, including ultrastructural damage, disruption of bioenergetic processes, altered cytological and weight indices, and behavioral responses, can be early signs of health disorders induced by CdO NPs, thus proving neurotoxic properties of the latter.

## Introduction

Industrial emissions of cadmium, which is used in various industries, contribute to the aggravation of the problem of chemical pollution of the environment and workplace air. Along with mercury and lead, cadmium is one of the most hazardous and widespread heavy metals (Genchi et al. 2020). Long-term clearance and multisystem toxicity of cadmium account for the development of dangerous diseases, including osteomalacia, renal and hepatic dysfunction, reproductive disorders, and damage to organs of the hematopoietic system (Tinkov et al. 2018).

Generation of nanoparticles during high-temperature operations and their targeted use in various fields of science, medicine, and technology are of special concern (Ghasempour et al. 2023). Unique physicochemical properties of nano-sized particles significantly increase their biological activity by facilitating their penetration though cell membranes (Sutunkova et al. 2020) and even the blood-brain barrier (Boyes and van Thriel 2020). When in cell, NPs can partially dissolve and release ions (Demir et al. 2020), induce oxidative stress, genotoxic effects, damage to organelles, disruption of their functioning (Apykhtina et al. 2018; Sutunkova et al. 2023), and cell death (Jorio 2016).

**Fig. 1:**
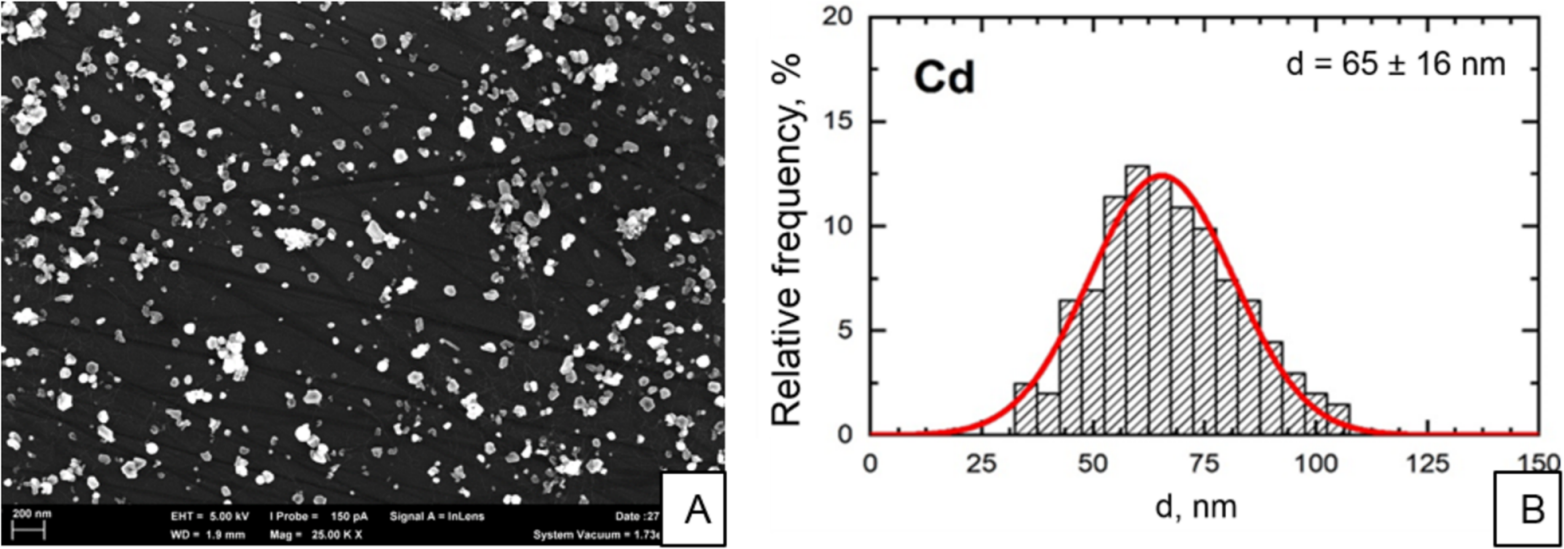
Description of cadmium oxide nanoparticles used in the experiment: **A** SEM image of suspended CdO NPs at 29,640x magnification; **B** size distribution of CdO NPs

Despite the published data available, the number of toxicity studies of nanoparticles of cadmium and its compounds is very limited and often represented by in vitro experiments (Demir et al. 2020; Apykhtina et al. 2018). However, scarce in vivo studies confirm danger of nanoscale cadmium metal particles by showing their hepatotoxic effects in mice (Li et al. 2018; Meng et al. 2019) and pathological pulmonary changes in rats (Katsnelson et al. 2017). In addition, it is worth noting that the vast majority of studies focused on cadmium sulfide NPs that are quite different in several important aspects, including biodistribution, reactogenicity, and generation. Yet, it is CdO NPs that are most often formed during high-temperature industrial processes like welding and smelting. These differences determine unique toxicokinetic profiles, thus necessitating targeted studies of CdO NPs.

When searching for the recent data on CdO NP toxicity published in 2020–2025 in PubMed, we found only one in vitro study of the cyto- and genotoxicity of CdO NPs (Demir et al. 2020) and one in vivo study conducted on Danio rerio fish (Vasanthakumaran et al. 2024). Insufficient understanding of the toxic impact of cadmium nanoparticles, coupled with the serious multisystem toxicity of this metal, determine the need to expand knowledge about possible toxic effects of cadmium nanoparticles and mechanisms of their exertion, enabling us to assess their potential health risks for industrial workers and develop a set of effective preventive measures.

## Materials and methods

### Generation and physicochemical characteristics of cadmium oxide nanoparticles (CdO NPs)

The stable suspension of cadmium oxide nanoparticles was prepared by laser ablation of metal cadmium targets (99.99%) in deionized water using an Fmark-20RL laser system of metal processing (Laser Technology Center, St. Petersburg, Russia) based on a pulsed ytterbium-doped fiber laser (pulse duration: 10 ns; repetion rate: 21 kHz; wavelength: 1,064 nm) at the Ural Center for Shared Use “Modern Nanotechnologies” at the Ural Federal University, Yekaterinburg, Russian Federation. This method has been regularly applied by our team and is described in detail elsewhere (Katsnelson et al. 2017).

The shape and size of particles were established by scanning electron microscopy using a Carl Zeiss Auriga® CrossBeam® Workstation, Germany. The size distribution function was obtained by statistical analysis of SEM images; the mean diameter of resulting cadmium oxide nanoparticles was 65 ± 16 nm (Fig. 1).

The suspension stability was judged by the zeta potential measured using the Zetasizer Nano ZS size analyzer (Malvern Panalytical, UK) and was found to be high (45 mV), which allowed the suspension to be safely concentrated to 0.5 mg/mL by controlled evaporation of the solvent without changing the size and chemical identity of nanoparticles.

### Experimental animals

The experiment was conducted on female outbred albino adult rats aged 3 to 4 months from our own breeding colony with the initial body weight of 204,65 ± 3,17 g.

We acknowledge the importance of considering sex differences in toxicokinetics and toxicodynamics of nanoparticles. The choice of female rats was attributed to their demonstration of a more pronounced inflammatory response to metal NPs in the airways (Ćurlin et al. 2021; Canup et al. 2024), which was important at the initial stages of the study and for identifying early predictors and mechanisms. This strategy is consistent with the principles for assessing risks in sensitive subpopulations of the U.S. Environmental Protection Agency (2023).

The rats were kept in a specially equipped vivarium room in compliance with the International Guiding Principles for Biomedical Research Involving Animals by CIOMS and ICLAS (2012).

The experiment was designed and conducted in accordance with the same document and approved by the Ethics Committee of the Yekaterinburg Medical Research Center for Prophylaxis and Health Protection in Industrial Workers (protocol No. 2 of April 20, 2020).

The animals were randomly divided into two groups (with/without Cd NP exposure) of 14 females each. The exposed rodents were administered 50 µl of CdO NP suspension at the concentration of 0.5 mg/mL intranasally into each nostril thrice a week during 6 weeks (18 administrations and 0.9 mg in total), while the control group was administered a similar volume of deionized water.

Since the maximum allowable concentration of CdO NPs has not been established to date, our dosage of 0.9 mg per rat over 6 weeks was chosen based on other experiments with nanoparticles and was in the low to moderate range in terms of exposure intensity.

A similar dose was used in yet another experiment of ours with PbO NPs (Sutunkova et al. 2022). Besides, a comparable dose was used by Wen et al. (2016) in their study of Ag NPs administered during 12 weeks.

The dose selection was also limited by the nanoparticle concentration in the suspension and the maximum permissible volume for intranasal administration to rats. The concentration and volume administered were technically feasible and standard for the intranasal route of administration to rats.

The thrice a week administration schedule was a compromise between the need for recurrent exposure and minimizing stress and damage to the animals. Daily administration could have been more stressful for the animals and might have increased the risk of local irritation and injury.

### Assessment of the animals

During the experiment, we weighed the animals weekly, registered the summation threshold index (STI) (Speransky 1975), and conducted a hole-board test to establish their exploratory behavior, locomotion, grooming, and defecation. Animal habituation to all testing procedures was carried out in accordance with standard protocols.

Following exposure cessation, prior to euthanasia, we prepared imprint smears of the nasal cavity (Sakhautdinova et al. 2020) and took samples of nasal washings for cytomorphological examination. We then carried out Romanowski-Giemsa staining of the cytological preparations and examined them using a Carl Zeiss microscope at 100x magnification.

We used isoflurane anesthesia as an adjuvant to ensure painless sacrifice of the rats by rapid decapitation, followed by autopsy, weighing of internal organs (liver, kidneys, spleen, brain, lungs, and heart) and recalculation of measured values per 100 g of body weight, blood sampling for biochemical testing, smear preparation, and complete blood count. We also evaluated the results of cytochemical testing, i.e., determination of activity of succinate dehydrogenase (SDH) of blood lymphocytes using p-nitrotetrazolium violet by counting the number of formazan granules in 50 cells to establish the bioenergetic metabolism (Narcissov 1969).

To avoid bias, blinding was ensured at all stages of work including behavioral and laboratory testing. The researchers observing the animals or evaluating the outcomes were unaware of group affiliation until the completion of data collection and statistical analysis.

### Electron microscopy

We examined specimens of the olfactory bulb and basal ganglia of the rats from both groups using scanning electron microscopy. Brain specimens were cut into small cubes of about 1 mm$^3$. Tissues were then fixed in 2.5 % glutaraldehyde in 0.1 M phosphate buffer (pH 7.4) for 20 h at 4 °C. Afterwards, the samples were post-fixed in 1% osmium tetroxide in 0.1 M phosphate buffer (pH 7.4) for 90 min, dehydrated in an ascending ethanol series, and embedded in Epon812 resin. Thin 70-nm sections were then cut using a Leica EM UC7 ultramicrotome (Leica Microsystems, Austria), placed on 200 mesh copper grids, and stained with uranyl acetate and lead citrate solutions.

To study morphological changes in myelin sheaths of axons and to assess the state of mitochondria in neurons, three to four series of five to six sections were made with an interval of at least 100 µm for each specimen, which were then visualized with a Hitachi REGULUS SU8220 scanning electron microscope in STEM mode.

An Ultim Extreme energy dispersive X-ray spectral (EDS) detector, a 100 mm$^2$ windowless version of Ultim (Oxford Instruments, UK), was used to identify CdO nanoparticles. The spectra were analyzed using Aztec software (Oxford Instruments, UK), and images were processed using ImageJ (US National Institutes of Health) and GIMP 2.8 (GNU Image Manipulation Program).

To determine the degree of mitochondrial damage after cadmium oxide nanoparticle exposure, we registered five consecutive stages of inner membrane transformation according to the classification by Sun et al. (2007). Morphotyping was performed by two researchers independently in order to reduce contribution of the human factor, after which the resulting percentage distribution of morphotypes was averaged for each type of tissue and animal.

**Table 1:** Body and organ weight measurements ($\bar{X} \pm S_x$)

Parameters	Groups of experimental animals	
	Control	CdO NP exposure
Pre-exposure body weight, g	205.36 ± 2.59	203.93 ± 3.75
Post-exposure body weight, g	243.21 ± 4.65	239.64 ± 5.92
Body weight gain, %	37.86 ± 3.39	35.71 ± 4.97
Relative liver weight, g/100 g BW	3.18 ± 0.09	3.29 ± 0.12
Relative kidney weight, g/100 g BW	0.54 ± 0.02	0.55 ± 0.01
Relative spleen weight, g/100 g BW	0.22 ± 0.01	**0.28 ** $\boldsymbol{\pm }$ 0.01*
Relative brain weight, g/100 g BW	0.81 ± 0.02	0.79 ± 0.02
Relative lung weight, g/100 g BW	0.60 ± 0.07	0.54 ± 0.02
Relative heart weight, g/100 g BW	0.31 ± 0.01	0.30 ± 0.01

* $p < 0.05$, based on the Student’s *t*-test

**Table 2:** Biochemical blood parameters measured in the experimental rats ($\bar{X} \pm S_x$)

Parameters	Groups of experimental animals	
	Control	CdO NP exposure
Plasma sulfhydryl (SH) groups, mmol/L	4.85 ± 1.00	5.18 ± 1.02
Reduced glutathione, µmol/L	33.77 ± 4.69	31.88 ± 3.23
Serum catalase, µmol/L	0.72 ± 0.10	0.81 ± 0.03
Serum malondialdehyde (MDA), µmol/L	51.54 ± 9.30	27.32 ± 9.05
Serum ceruloplasmin, mg/%	80.61 ± 1.82	**64.82 ** $\boldsymbol{\pm }$ 4.11*
SDH activity, number of formazan granules per 50 lymphocytes	583.70 ± 6.24	**459.35 ** $\boldsymbol{\pm }$ 6.17*
Serum myelin basic protein, ng/mL	2.44 ± 0.43	2.12 ± 0.24

* $p < 0.05$, based on the Student’s *t*-test

### Statistical data analysis

The statistical analysis of data obtained in behavioral tests, biochemical blood tests, and complete blood counts were carried out using the Student’s *t*-test ($p \le 0.05$), cytology—using the Mann–Whitney U-test, and electron microscopy—using the Mann–Whitney U-test and the Pearson’s goodness-of-fit test ($p \le 0.05$). Behavioral test scores were calculated and normalized by bringing control values to unity.

The mitochondrial damage index was calculated by ranking the mitochondrial types, where 1 was taken as the normal morphotype, 0.5 as normal vesicular, 0.25 as vesicular, vesicular swollen, and 0 as swollen. The relative mitochondrial index was then derived by normalizing these results to the control group (dividing them by the median value of controls) and statistical significance was established using the Mann–Whitney U-test.

### Limitations

Limitations of the study included the use of a single animal model (female rats only) and exposure dose. The design did not account for potential sex differences and did not establish dose-response relationships. The screening nature of the study also limited the mechanistic analysis.

## Results

### Analysis of experimental data on the general toxic effect

Table  1 shows a statistical increase in the relative spleen weight in the animals exposed to CdO NPs compared to the controls.

We observed a statistical decrease in the ceruloplasmin level in blood serum and succinate dehydrogenase (SDH) activity tested in blood lymphocytes of the rats following the CdO NP exposure (Table 2).

The results of testing hematological parameters in the animals revealed no adverse changes in the exposed rats that would be statistically different from those in the controls, yet we observed a noticeable increase in the proportion of reticulocytes (Table 3).

**Table 3:** Hematological indices measured in the experimental rats ($\bar{X} \pm S_x$)

Parameters	Groups of experimental animals	
	Control	CdO NP exposure
Red blood cell count, $10^{12}$ cells/L	6.73 ± 0.13	6.61 ± 0.09
Reticulocytes, ‰	14.92 ± 2.01	20.00 ± 1.86
Hemoglobin, g/L	148.46 ± 2.08	146.29 ± 2.16
Hematocrit, %	18.12 ± 0.24	17.58 ± 0.25
Mean corpuscular volume, µm$^{3}$	53.93 ± 0.65	53.21 ± 0.58
Mean corpuscular hemoglobin, $10^{-12}$ g	22.10 ± 0.35	22.16 ± 0.40
Mean corpuscular hemoglobin concentration, g/L	409.62 ± 2.80	416.50 ± 4.91
Red cell distribution width, ±	13.64 ± 0.17	14.01 ± 0.18
Leukocytes, $10^6$ mL	6.88 ± 0.53	7.21 ± 0.70
Basophils, $10^6$ mL	0.00 ± 0.00	0.00 ± 0.00
Eosinophils, $10^6$ mL	0.15 ± 0.05	0.27 ± 0.06
Banded neutrophils, $10^6$ mL	0.08 ± 0.01	0.07 ± 0.01
Segmented neutrophils, $10^6$ mL	1.14 ± 0.11	1.27 ± 0.15
Monocytes, $10^6$ mL	0.40 ± 0.05	0.40 ± 0.04
Lymphocytes, $10^6$ mL	5.10 ± 0.38	5.20 ± 0.50
Basophils, %	0.00 ± 0.00	0.00 ± 0.00
Eosinophils, %	2.15 ± 0.52	3.64 ± 0.68
Banded neutrophils, %	1.15 ± 0.10	1.00 ± 0.00
Segmented neutrophils, %	16.46 ± 0.97	17.50 ± 0.88
Monocytes, %	5.62 ± 0.38	5.64 ± 0.32
Lymphocytes, %	74.62 ± 1.34	72.21 ± 0.79
Platelet count, $10^6$ mL	838.62 ± 49.56	830.00 ± 56.01
Mean platelet volume, µm$^{3}$	5.97 ± 0.10	5.99 ± 0.14
Plateletcrit, %	0.25 ± 0.01	0.25 ± 0.02
Platelet distribution width, %	12.67 ± 0.28	12.26 ± 0.30

**Fig. 2:**
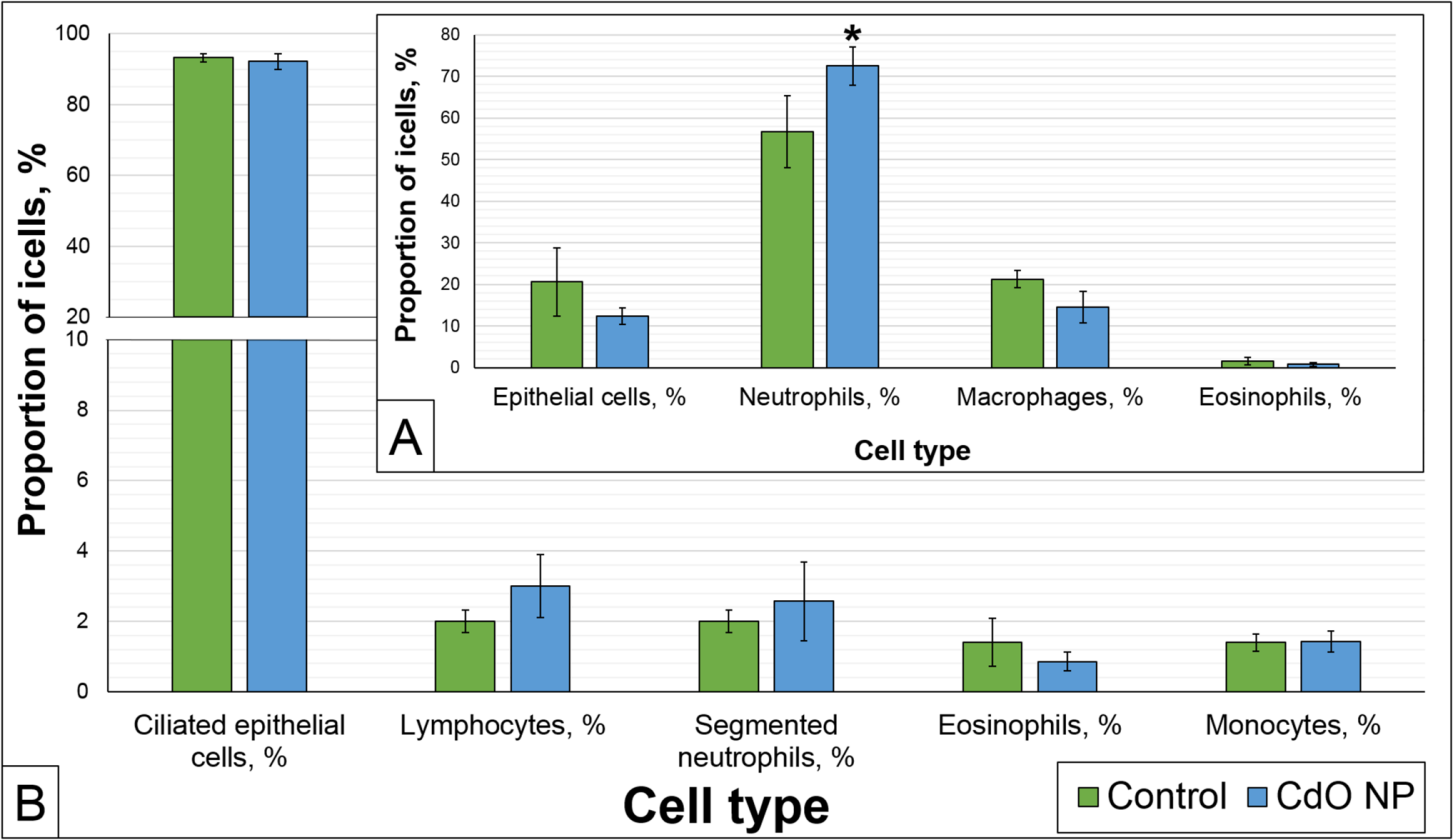
Cytological indices of rhinocytological washings (**A**) and imprint smears of the nasal cavity (**B**) of the experimental rats. * $p< 0.05$, based on the Mann–Whitney U-test

**Fig. 3:**
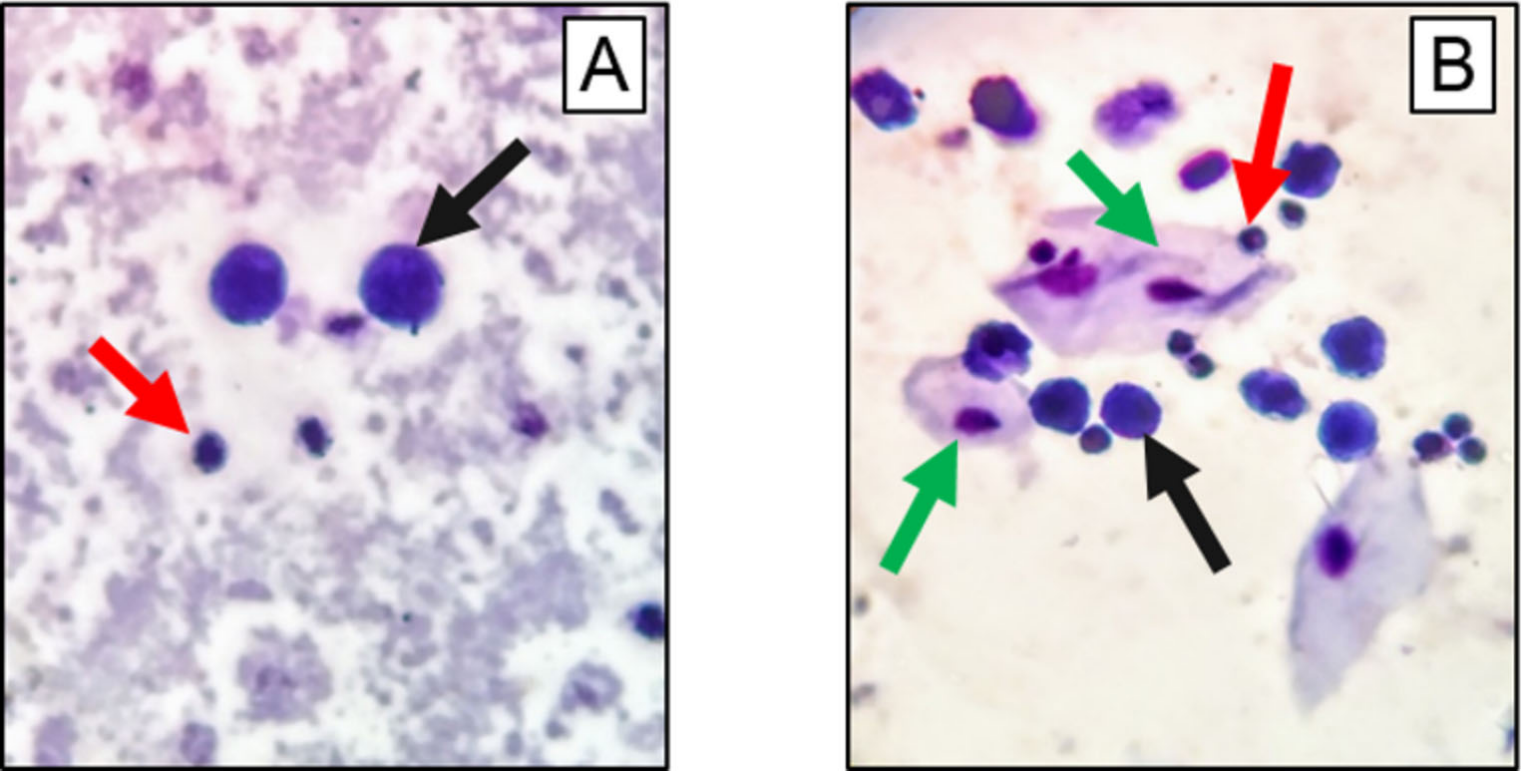
Micrographs of preparations of rhinocytological specimens of the CdO NP exposed rats showing **A** macrophages and segmented neutrophils; **B** epithelial cells, macrophages, and segmented neutrophils; Romanowsky-Giemsa staining, 100x magnification. Black, red, and green arrows indicate macrophages, segmented neutrophils, and epithelial cells, respectively

### Cytological changes

The analysis of the ratio of different cell types in rhinocytological washings and smears showed almost comparable results in both groups. Intranasal administration of CdO NPs only caused a statistical increase in the proportion of neutrophils, a noticeable, yet insignificant, decrease in the proportion of macrophages in washings (Figs. 2 and 3), and an increase in segmented neutrophils and lymphocytes in imprint smears (Fig. 2).

### Changes in the nervous system

**Fig. 4:**
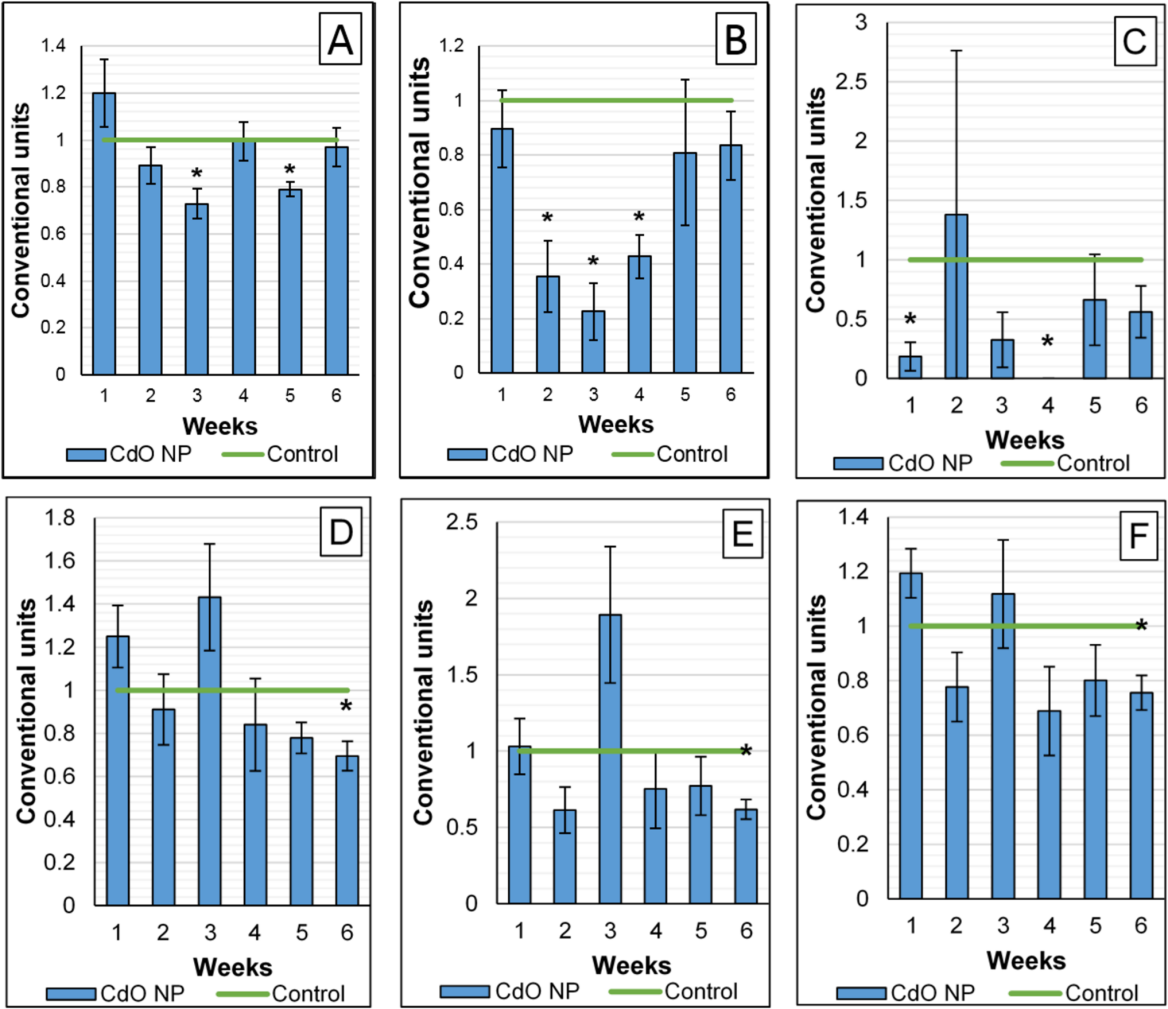
Behavioral test results: **A** summation threshold index; hole-board test: **B** head lifts, **C** rearing, **D** head dipping into holes, **E** hole sniffing, and **F** general locomotion. * $p< 0.05$, based on the Mann–Whitney U-test

The results of the behavioral tests conducted weeky during the CdO NP exposure period showed a decrease in all indices by week 3 in both groups, which was then leveled out (Fig. 4A–C). For a number of parameters assessed in the hole-board test, however, a significant decrease in exloratory behavior and general locomotion by the end of the experiment was registered in the exposed rodents (Fig. 4D–F).

**Fig. 5:**
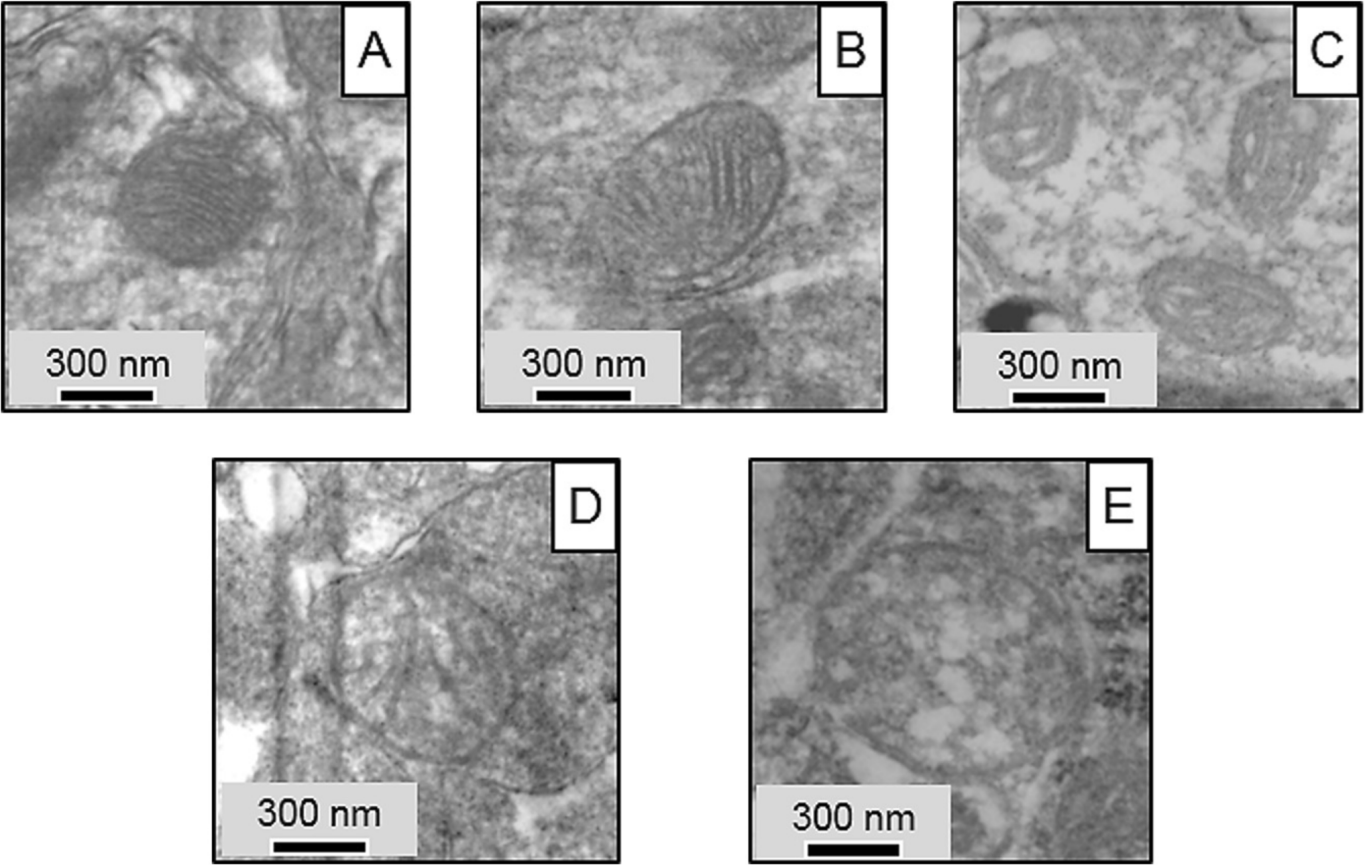
SEM images of **A** normal, **B** normal vesicular, **C** vesicular, **D** vesicular swollen, and **E** swollen mitochondrial morphologies found in rat tissues of both studied groups

**Fig. 6:**
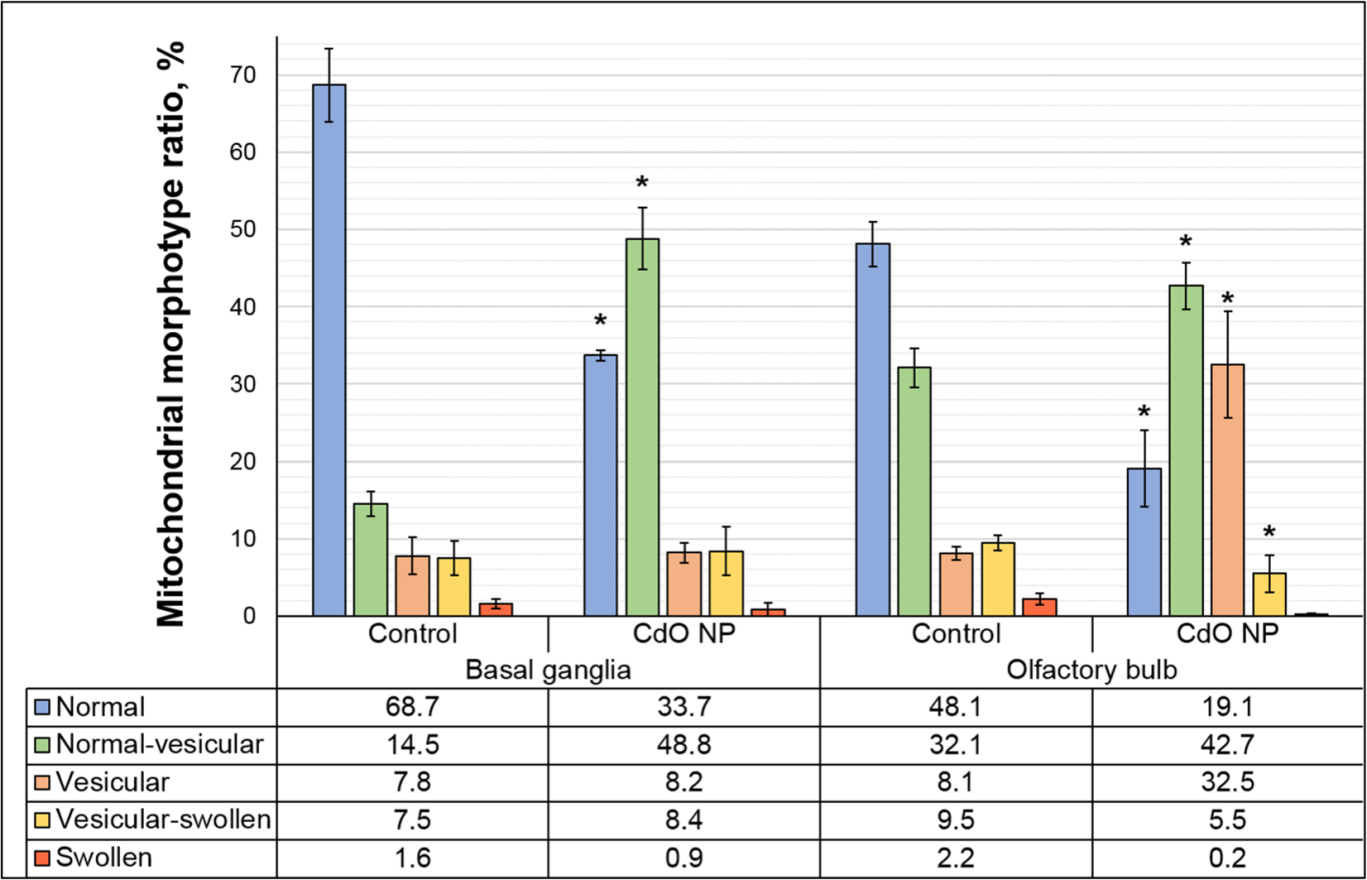
The ratio of mitochondrial morphotypes in neurons of the experimental rats. Notes: Values are given as the arithmetic mean of the proportion of mitochondria of the total number of detected neuronal mitochondria. * $p< 0.05$, based on the Mann–Whitney U-test

### Electron microscopy findings

Electron microscopy and high-quality EDS revealed no deposition of CdO NPs in cells of olfactory bulbs or basal ganglia in the experimental rats. However, during the assessment of the state of mitochondria of the neurons in the olfactory bulbs and basal ganglia of the experimental animals according to the classification proposed by Sun et al. (2007), we identified the following stages of change in the topology of the inner mitochondrial membrane: normal, normal vesicular, vesicular, vesicular swollen, and swollen. Image analysis of micrographs of neuronal mitochondria proved the presence of all the above types in the specimens (Fig. 5). The images also showed signs of damage to the double membrane contour, damage to the mitochondrial cristae, and matrix clearing.

Evaluation of all mitochondria detected in the ultrathin section helped classify the organelles under study based on the topology of the inner membrane and then make a calculation for each mitochondrial morphological type (Fig. 6).

The use of the Pearson’s goodness-of-fit test allowed us to statistically confirm the above differences in the distribution of mitochondrial morphotypes between the exposed and control animals ($\chi ^2$ BG (4; 0.05) = 994.03; $p \le 0.0001$; $\chi ^2$ OB (4; 0.05) = 979.88; $p \le 0.0001$), as well as between the regions of the brain ($\chi ^2$ (4; 0.05) = 8064, $p \le 0.0001$).

Following CdO NP administration, we observed a statistically significant decrease in the proportion of the normal morphotype by 29.0% and an increase in the proportion of the normal vesicular morphotype of mitochondria by 10.6% in the basal nuclei of the exposed rats compared to the controls. Similar trends were observed in the olfactory bulb specimens: the proportion of normal mitochondria statistically decreased by 35.0%, while those of normal vesicular and vesicular mitochondria increased by 34.3% and 24.4% in the exposed rodents, respectively.

**Fig. 7:**
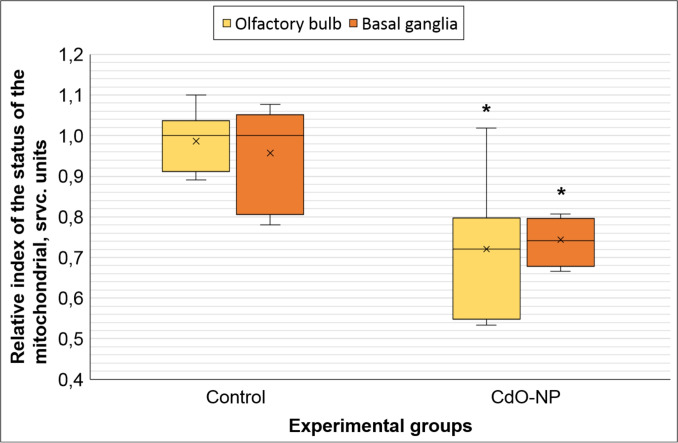
Relative index of the status of the mitochondrial apparatus in the experimental rats. * $p< 0.05$, based on the Mann–Whitney U-test

The results of comparing the relative indicator reflecting the state of the mitochondrial apparatus showed the absence of statistically significant differences between the indicators of two different areas of the brain in the control rats, while the exposed animals demonstrated a statistically significant decrease in this parameter in both areas of the brain compared to the controls (Fig. 7).

**Fig. 8:**
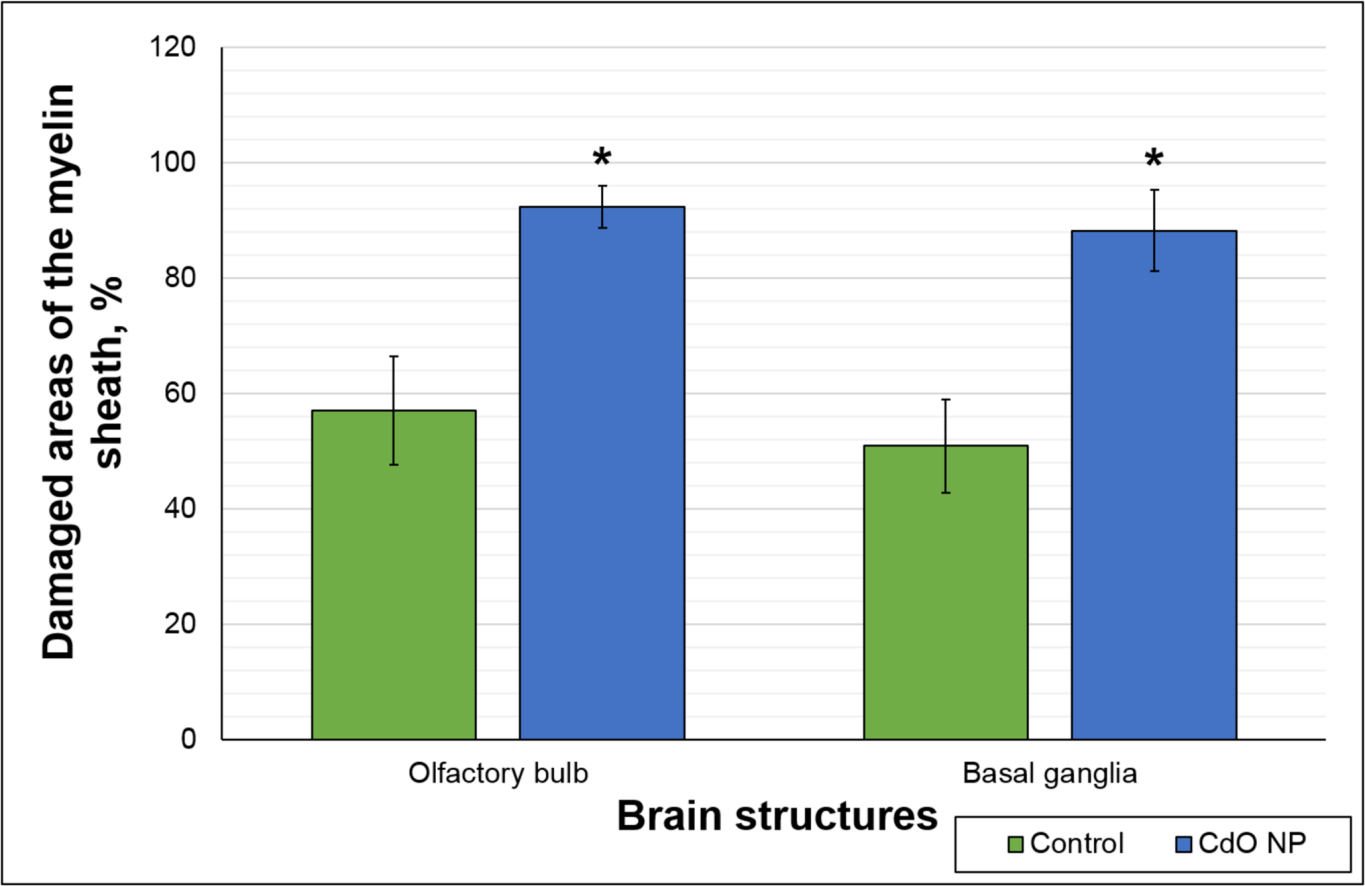
The proportion of damage to the ultrastructure of the myelin sheaths of axons in the brain regions of the experimental rats. * $p< 0.05$, based on the Mann–Whitney U-test

**Fig. 9:**
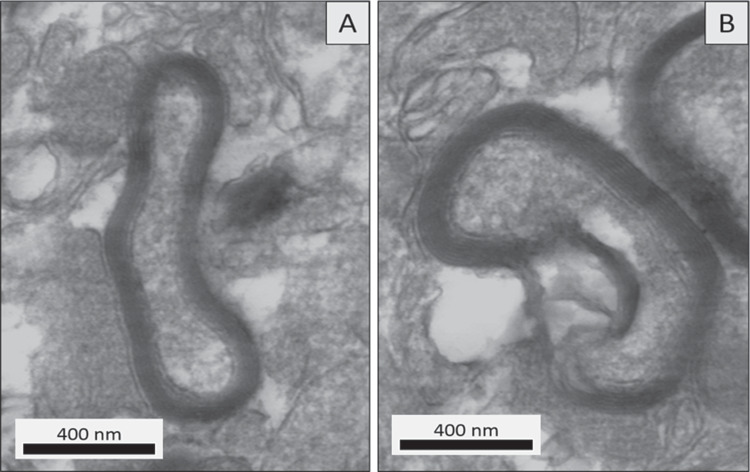
STEM images of the normal (**A**) and damaged (**B**) myelin sheath

Ultrastructural examination of the myelin sheath revealed a statistically significant increase in the amount of damage to the myelin sheath of axons in tissues of the olfactory bulbs (by 35.3%) and basal ganglia (by 37.3%) in the exposed animals compared to the controls (Figs. 8 and 9).

## Discussion

The study showed that the exposure to CdO NPs induced an increase in the splenic weight in the absence of expected shifts in this parameter for the liver and kidneys, which may indicate an uneven distribution of these NPs in the rat body following intranasal administration, as well as the fact that toxic effects of nano-sized cadmium are likely to differ from the effects caused by its other forms (e.g., soluble salts) due to the specifics of interaction of the latter with living systems attributable to their unique physicochemical properties (Nguyen et al. 2019).

Intranasal administration of CdO NPs altered behavioral responses, but not hematological or cytological characteristics. We assume that only early signs of cadmium poisoning at the organismal level are manifested at such a level and duration of exposure. Thus, changes in blood parameters were statistically insignificant, but some activation of erythropoiesis expressed by a noticeable increase in the proportion of reticulocytes was noted. The results of the cytological examination of rhinocytological specimens indicate nonspecific resistance to the toxicant manifested by an increased neutrophil count, which, in the absence of systemic inflammatory shifts in the blood (Table 3), indicate local inflammation.

A more pronounced response of the nervous system can, in turn, be attributed to nanoparticle translocation upon intranasal and inhalation route of administration (Kao et al. 2012; Minigalieva et al. 2023). The observed decrease in the behavioral test indices by week 3 in both groups may be a response to stress caused by manipulations and then relieved by adaptation. In the exposed rats, however, certain parameters, such as hole dipping and sniffing and general locomotion, all reflecting exploratory behavior of the animals, were persistently suppressed and statistically different from those in the controls. Such disorders indicate a pronounced adverse effect of cadmium oxide nanoparticles on the nervous system confirmed by the results of studying the ultrastructure of neurons in the olfactory bulbs and basal ganglia of the exposed rodents. The observed myelin disruption in the latter (Fig. 9), coupled with myelin deterioration leading to microglia activation via release of inflammatory mediators (Elder et al. 2006), accounts for improper axonal signal transmission and induces the behavioral deviations registered. Similar disorders have been repeatedly observed by our team in experimental studies of toxic effects of various metal/metal oxide nanoparticles with different routes of their administration (Dumková et al. 2017), allowing us to conclude on the neurotoxic effect intrinsic to them.

Adverse shifts in the nervous system may also be supported by the observed changes in the mitochondrial transport in neurons. These organelles are known to play an important role in apoptosis (Sun et al. 2007). The differences noted in the ratio of mitochondrial morphotypes in basal ganglia and olfactory bulb tissues in both groups may indicate the specificity of this parameter even within a single organ, thus emphasizing the importance of precise location of the study zone. Even so, in both zones under study, we observed a significant decrease in the proportion of normal mitochondria and an increase in that of normal vesicular ones in neurons of the exposed rats compared to the controls. Besides, a statistical increase in the proportion of vesicular mitochondria in neurons of the olfactory bulb of the exposed animals supports uneven distibution of nanoparticles in the rat brain during migration along the olfactory nerve (Dumková et al. 2017). Nevertheless, the results of comparing the mitochondrial profile of the experimental animals confirm the comparable negative impact of CdO NPs on the mitochondrial apparatus of cells of both olfactory bulbs and basal ganglia (Fig. 7). However, the destructive processes in mitochondria induced by this level of CdO NP exposure may be reversible, which is confirmed by a low percentage of swollen morphotype mitochondria (<2.5%) indicating a short period of time since the release of cytochrome, so in case of timely exposure cessation, cells can restore the mitochondrial function providing that the integrity of the membranes and DNA is preserved (Shiozaki and Shi 2004; Perkins et al. 2009).

Nevertheless, the defects of the inner mitochondrial membrane found by electron microscopy lead to a failure in the functioning of the enzyme systems involved in a number of important bioenergetic reactions, which is partly confirmed by a reliable decrease in SDH activity in blood lymphocytes of the exposed rats (Table 2). In addition, disruption of normal functioning of cell mitochondria entails increased oxidative processes in cells, as evidenced by a statistical decrease in the concentration of ceruloplasmin in blood plasma of the animals administered CdO NPs intranasally (Table 2). The decrease in this indicator is also facilitated by a known competition of cadmium and copper, which are part of this metallothionein, for binding sites (Ohta et al. 2002). In total, the observed changes can act as markers of redox imbalance and give evidence of the increased oxidative stress in the animal organism, which, in turn, is one of the main factors stimulating the development of a wide range of nervous diseases.

## Conclusions

Our findings show a number of general toxicity manifestations at the organismal level and a clear neurotoxic effect of subchronic intranasal exposure to CdO nanoparticles in a total dose of 0.9 mg per rat.

The observed shifts in exploratory behavior and locomotion of the exposed animals, supported by the registered changes in the ratio of mitochondrial morphotypes, an increase in the number of axons with damage to the myelin sheath in the basal ganglia and tissues of the olfactory bulb, along with a decrease in the ceruloplasmin concentration and SDH activity, may indicate a disruption in the functioning of mitochondria, entailing a cascade of oxidative reactions leading to cell apoptosis.

The negative changes identified at all levels of organization, including ultrastructural defects and behavioral disturbances, indicate that CdO NPs can exhibit neurotoxic properties. They can also be early signs of health disorders and serve as a basis for further development of methods for early diagnosis of cadmium poisoning.

## Data Availability

All source data for this work (or generated in this study) are available upon reasonable request.
